# Supplementary material for: A conserved filamentous assembly underlies the structure of the meiotic chromosome axis
Source: eLife. 2019 Jan 18;8:e40372. doi: 10.7554/eLife.40372 (PMC6349405; doi:10.7554/eLife.40372)
Supplement: Supplementary file 1. [file elife-40372-supp1.docx]

**Supplementary File 1 - Data Collection and Refinement Statistics**

|  | ***Mm* SYCP3^CC^ P2_1_** | ***Mm* SYCP3^CC^ P1** |
| --- | --- | --- |
| **Data collection** |  |  |
| Synchrotron/Beamline | APS 24ID-C | APS 24ID-C |
| Resolution (Å) | 50 – 2.6 | 72 – 2.3 |
| Wavelength (Å) | 0.9793 | 0.9200 |
| Space Group | P2_1_ | P1 |
| Unit Cell Dimensions (a, b, c) Å | 45.80, 49.39, 150.26 | 45.87, 52.28, 75.60 |
| Unit cell Angles (α,β,γ) ° | 90, 90.80, 90 | 95.16, 103.69, 110.54 |
| *I*/σ (last shell) | 5.8 (0.8) | 10.0 (1.5) |
| ^b^ *R*_meas_ (last shell) | 0.132(1.66) | 0.088 (0.967) |
| ^c^ CC_1/2_, last shell | 0.783 | 0.849 |
| Completeness (last shell) % | 98.7 (98.7) | 92.0 (92.6) |
| ^d^ Number of reflections | 140357 | 190603 |
| *unique* | 40144 | 51353 |
| Multiplicity (last shell) | 3.5 (3.5) | 3.7 (3.7) |
| **Refinement** |  |  |
| Resolution (Å) | 50 – 2.6 | 72 – 2.3 |
| ^d^ No. of reflections | 39596 | 50966 |
| *working* | 37454 | 48415 |
| *free* | 2142 | 2551 |
| ^e^ *R*_work_ (last shell) % | 25.37 (44.70) | 28.71 (38.17) |
| ^e^ *R*_free_ (last shell) % | 32.30 (50.80) | 33.10 (40.58) |
| **Structure/Stereochemistry** |  |  |
| Number of atoms | 4123 | 4119 |
| *solvent* | 0 | 10 |
| r.m.s.d. bond lengths (Å) | 0.009 | 0.008 |
| r.m.s.d. bond angles (°) | 0.930 | 0.933 |
| ^f^ PDB ID | 6DD8 | 6DD9 |
| ^g^ SBGrid Data Bank ID | 583 | 584 |

^b^ *R*_meas_ = ∑_h_ [ √(*n*/(*n*-1)) ∑_j_ [*I*_hj_ - 〈*I*_h_〉] / ∑_hj_ 〈*I*_h_〉, where *I*_hj_ is a single intensity measurement for reflection h, 〈*I*_h_〉 is the average intensity measurement for multiply recorded reflections, and *n* is the number of observations of reflection h.

^c^ CC_1/2_ is the Pearson correlation coefficient between the average measured intensities of two randomly-assigned half-sets of the measurements of each unique reflection (Karplus and Diederichs 2012).

^d^ Reflection counts in scaling and refinement are unmerged (*F*(+) and *F*(-) treated as separate reflections).

^e^ *R*_work, free_ = ∑||F_obs_| – |F_calc_||/|F_obs_|, where the working and free *R*-factors are calculated using the working and free reflection sets, respectively.

^f^ Coordinates and structure factors have been deposited in the RCSB Protein Data Bank (www.pdb.org) with the noted accession numbers.

^g^ Diffraction data have been deposited with the SBGrid Data Bank (https://data.sbgrid.org) with the noted accession numbers.
