# Supplementary material for: A conserved filamentous assembly underlies the structure of the meiotic chromosome axis
Source: eLife. 2019 Jan 18;8:e40372. doi: 10.7554/eLife.40372 (PMC6349405; doi:10.7554/eLife.40372)
Supplement: Supplementary file 2. [file elife-40372-supp2.docx]

**Supplementary File 2 – SYCP3-SYCP2 crosslinks**

| **SYCP3 Residue** | **SYCP2 Residue** | **# observations** |
| --- | --- | --- |
| 88 | 1371 | 12 |
| 88 | 1379 | 3 |
| 88 | 1455 | 2 |
| 88 | 1465 | 1 |
| 88 | 1503 | 2 |
| 97 | 1371 | 1 |
| 97 | 1495 | 8 |
| 97 | 1503 | 4 |
| 101 | 1371 | 5 |
| 101 | 1455 | 6 |
| 101 | 1495 | 20 |
| 112 | 1371 | 4 |
| 159 | 1371 | 3 |
| 159 | 1433 | 21 |
| 159 | 1437 | 8 |
| 159 | 1465 | 2 |
| 173 | 1371 | 3 |
| 173 | 1433 | 203 |
| 173 | 1445 | 4 |
| 176 | 1371 | 1 |
| 176 | 1433 | 5 |
| 205 | 1371 | 10 |
| 205 | 1379 | 13 |
| 205 | 1390 | 32 |
| 205 | 1455 | 2 |
| 213 | 1371 | 22 |
| 213 | 1379 | 4 |

Yellow shading indicates an interaction observed at least 8 times, and orange shading indicates an interaction observed at least 20 times (**Figure 4F**, **Figure 4 – Figure Supplement 5**).
