# Supplementary material for: A conserved filamentous assembly underlies the structure of the meiotic chromosome axis
Source: eLife. 2019 Jan 18;8:e40372. doi: 10.7554/eLife.40372 (PMC6349405; doi:10.7554/eLife.40372)
Supplement: Supplementary file 3. [file elife-40372-supp3.docx]

**Supplementary File 3 – SYCP3-SYCP3 and SYCP2-SYCP2 crosslinks**

| **SYCP3-SYCP3 Crosslinks** | | |
| --- | --- | --- |
| **SYCP3 Residue** | **SYCP3 Residue** | **# observations** |
| 90 | 88 | 3 |
| 101 | 90 | 32 |
| 112 | 88 | 2 |
| 112 | 101 | 13 |
| 149 | 159 | 13 |
| 159 | 88 | 4 |
| 159 | 101 | 2 |
| 176 | 159 | 6 |
| 205 | 88 | 2 |
| 205 | 101 | 5 |
| 205 | 213 | 9 |
| 214 | 88 | 6 |
| 214 | 90 | 9 |
| 214 | 101 | 40 |
| 229 | 205 | 2 |

| **SYCP2-SYCP2 Crosslinks** | | |
| --- | --- | --- |
| **SYCP2 Residue** | **SYCP2 Residue** | **# observations** |
| 1379 | 1371 | 54 |
| 1379 | 1503 | 3 |
| 1390 | 1371 | 2 |
| 1413 | 1433 | 1 |
| 1433 | 1371 | 1 |
| 1433 | 1449 | 4 |
| 1437 | 1449 | 14 |
| 1449 | 1371 | 3 |
| 1455 | 1371 | 4 |
| 1455 | 1465 | 9 |
| 1465 | 1371 | 4 |
| 1495 | 1503 | 1 |
| 1503 | 1371 | 3 |

Yellow shading indicates an interaction observed at least 8 times, and orange shading indicates an interaction observed at least 20 times (**Figure 4 – Figure Supplement 5**).
