# Supplementary material for: A conserved filamentous assembly underlies the structure of the meiotic chromosome axis
Source: eLife. 2019 Jan 18;8:e40372. doi: 10.7554/eLife.40372 (PMC6349405; doi:10.7554/eLife.40372)
Supplement: Supplementary file 5. [file elife-40372-supp5.docx]

**Supplementary File 5 - Yeast Strains**

| **Strain Name** | ***MAT*** | **Genotype** | **Reference** |
| --- | --- | --- | --- |
| JLY106 (RKY1145) | a | *leu2∆hisG his4-x ura3 lys2 ho::LYS2* (SK1) | (de los Santos and Hollingsworth, 1999; Hollingsworth et al., 1995) |
| JLY107 (S2683) | α | *leu2-k arg4-Nsp ura3 lys2 ho::LYS2* (SK1) | (de los Santos and Hollingsworth, 1999; Hollingsworth et al., 1995) |
| *KDC602 | a/α | Diploid from mating JLY106 with S2683 | this work |
| KDC624 | a | JLY106 + *RED1(1-362)-6xHis-3xHA::KanMX6* | this work |
| KDC626 | α | S2683 + *RED1(1-362)-6xHis-3xHA::KanMX6* | this work |
| KDC649 | a/α | Diploid from mating KDC624 with KDC626 | this work |
| KDC676 | a | JLY106 + *RED1(1-362)-6xHis-3xHA::KanMX6 hop1Δ::LEU2* | this work |
| KDC675 | α | S2683 + *RED1(1-362)-6xHis-3xHA::KanMX6 hop1Δ::LEU2* | this work |
| KDC677 | a/α | Diploid from mating KDC676 with KDC675 | this work |
| KDC633 | a | JLY106 + *RED1(1-734)-ZrRED1(707-798)::KanMX6* | this work |
| KDC635 | α | S2683 + *RED1(1-734)-ZrRED1(707-798)::KanMX6* | this work |
| *KDC653 | a/α | Diploid from mating KDC633 with KDC635 | this work |
| KDC636 | a | JLY106 + *RED1(1-734)-ZrRED1(707-791)::KanMX6* | this work |
| KDC638 | α | S2683 + *RED1(1-734)-ZrRED1(707-791)::KanMX6* | this work |
| *KDC654 | a/α | Diploid from mating KDC636 with KDC638 | this work |
| KDC738 | a | JLY106 + *RED1(1-734)-ZrRED1(707-798 I715R)::KanMX6* | this work |
| KDC739 | α | S2683 + *RED1(1-734)-ZrRED1(707-798 I715R)::KanMX6* | this work |
| *KDC740 | a/α | Diploid from mating KDC738 with KDC739 | this work |

*asterisks indicate four strains used for spore viability and chromosome spread assays.
